# Establishing a controlled hookworm human infection (CHHI) model for Africa: A report from the stakeholders meeting held in Lambaréné, Gabon, November 10–11, 2019

**DOI:** 10.1186/s13690-021-00650-z

**Published:** 2021-07-05

**Authors:** Ayodele Alabi, Mosarrof Hussain, Marie-Astrid Hoogerwerf, Christine Ndong Mengome, Moses Egesa, Emmanuella Driciru, Linda J. Wammes, Yvonne C. M. Kruize, Erliyani Sartono, Ayola Akim Adegnika, Peter G. Kremsner, Maria Yazdanbakhsh, Selidji Todagbe Agnandji

**Affiliations:** 1 Centre de Recherches Médicales de Lambaréné, BP242, Lambaréné, Gabon; 2 Department of Parasitology, Leiden University Medical Center, Albinusdreef 2, 2333 ZA Leiden, The Netherlands; 3 Department of Infectious Diseases, Leiden University Medical Center, Leiden, The Netherlands; 4 Medical Research Council/Uganda Virus Research Institute and London School of Hygiene & Tropical Medicine Uganda Research Unit, P.O. Box 49, Entebbe, Uganda; 5 Uganda Virus Research Institute, P.O. Box 49, Entebbe, Uganda; 6 Department of Medical Microbiology, Leiden University Medical Center, Leiden, The Netherlands; 7 Institute of Tropical Medicine, University of Tübingen, Tübingen, Germany; 8 German Center for Infection Research (DZIF), Tübingen, Germany; 9 Fondation pour la Recherche Scientifique, 72 BP45 Cotonou, Bénin

**Keywords:** Controlled human infection model, *Necator americanus*, Vaccine development, Gabon, The Netherlands

## Abstract

**Background:**

Hookworm is a major contributor to worldwide disease burden with over 230 million people infected. It has been identified as one of the Neglected Tropical Diseases that can be controlled and even eliminated through mass drug administration and other effective interventions. Mathematical models have shown that hookworm can only be eliminated via a vaccine. Controlled Hookworm Human Infection (CHHI) models can facilitate rapid development of vaccines and drugs.

**Methods:**

As a first step towards the establishment of CHHI in Africa, we held a stakeholders meeting in Lamberene, Gabon from 10 to 11 November 2019.

**Results:**

Discussions revolved around the roles of the different regulatory institutions concerned; the need to strengthen existing regulatory capacity and the role of legislation; creating Gabon-specific ethical guidelines to govern Controlled Human Infection (CHI) studies; development of a study protocol; consideration of cultural and social peculiarities; the need for regular joint review meetings between interested parties throughout the process of protocol implementation; and participant compensation. Moreover, operational considerations concerning the introduction of CHHI in Gabon include the use of the local strain of hookworm for the challenge infections, capacity building for the local production of challenge material, and the establishment of adequate quality assurance procedures.

**Conclusion:**

The workshop addressed several of the anticipated hurdles to the successful implementation of CHHI in Gabon. It is our aim that this report will stimulate interest in the implementation of this model in the sub-Saharan African setting.

**Supplementary Information:**

The online version contains supplementary material available at 10.1186/s13690-021-00650-z.

## Background

Hookworm infects around 230 million people worldwide [1], and is hyperendemic in several sub-Saharan countries, some of which have a disease prevalence of a third of the pediatric population [2]. Subsequently, these may result in up to 40% reduction in future wage-earning [3, 4], perpetuating the poverty of the infected. The global economic burden of hookworm infection is estimated to cost between $7.5 to $138.9 billion annually [5]. In the Central African region, Gabon is estimated to have the highest prevalence of hookworm infection at 26% [2] (Table 1).

**Table 1:** Population-adjusted prevalence of Hookworm from 2000 onwards and annual anthelmintic treatment needs in the Central African region

	Population aged < 20 years (1000s)	Prevalence of hookworm (%)	Number of anthelmintic doses for school-aged children (1000s)^**a**^
Cameroon	9199	9·9 (8·3–11·3)	3340 (3094–3550)
Central African Republic	2037	15·5 (12·1–19·9)	512 (388–660)
Chad	6405	7·4 (5·6–10·2)	415 (214–689)
Congo	1869	12·9 (7·6–34·5)	461 (203–942)
DR Congo	37 088	17·9 (15·5–21·3)	15 551 (13 586–17 628)
Equatorial Guinea	273	11·2 (6·0–21·1)	125 (82–188)
Gabon	592	26·0 (12·9–40·6)	347 (262–420)

**Adapted from Dimitrios-Alexios Karagiannis-Voules et al.** [2]

^**a**^ **WHO definition, age 5–14 years**

Preventative measures such as the mass drug administration of anthelmintic agents i.e. albendazole have already been undertaken however their efficacy is questionable [6]. The WHO had set the target to implement annual or semi-annual preventive chemotherapy for preschool and school-aged children in endemic areas with an overall coverage of at least 75% by 2020 [7]. A total of 483,207 children were deemed to be in need of preventive chemotherapy against Soil Transmitted Helminth Infections (STHIs) in Gabon in 2018 [8]. Indeed, of the 320,000 school-aged children (SAC) at risk in Gabon in 2018, only 120,000 (39%) received chemotherapy, in comparison to 50,000 (19%) that had received chemotherapy in 2016. However effective coverage (> = 75% of SAC) occurred at only 10% of implementation units.

Periodical deworming, despite numerous success stories [9, 10], is increasingly viewed as unfavorable because of high reinfection rates and the looming threat of anthelmintic resistance [11, 12]. Hence, novel anthelmintic drugs and vaccines are needed to add to hookworm control tools. Controlled human infection (CHI) models are a rapidly scalable approach used to establish product safety and proof of efficacy, allowing for quick selection of promising drug and vaccine candidates for phase II or III clinical trials [13, 14].

### Vaccine development for hookworm disease

The ideal anthelmintic control strategy would involve a multimodal approach comprising a combination of complementary tools such as drug therapy, a sanitation strategy, and the development of a vaccine. Extensive modelling indicates that along with annual MDA programs, the vaccination of 9–12 month old infants (alongside EPI), with a booster vaccine in those > 15 years, would best reduce disease prevalence and morbidity [15].

A human hookworm vaccine has been developed that is a combination of two recombinant antigens known as *Na*-GST-1 and *Na*-APR-1 [15]. Challenge studies conducted in laboratory animals were used to provide proof-of-concept of its efficacy [16]. Phase 1 clinical trials in healthy Gabonese adults was shown to be safe and to induce Immunoglobulin G to each antigen [17]. However, rNaGST-1, which showed highly neutralizing immunogenicity in mice challenge trials, has shown only negligible neutralizing immunogenicity in humans [18, 19]. All of the available animal models for hookworm do not replicate natural human hookworm infection; thus limiting the understanding of the disease including the human-host interactions that are essential in characterizing host responses, and mechanisms of pathogen evasion.

An Australian group has begun investigating the safety and tolerability of an attenuated live hookworm vaccine (ACTRN12617001007325).

### The controlled human infection model

A total of 15 controlled hookworm human infection studies involving 194 volunteers have been conducted in the USA, UK, Australia and the Netherlands (Additional file 1). Hoogerwerf and colleagues at the Leiden University Medical Centre (LUMC) have continued with the development of a controlled human infection model for *N. americanus* (CHHI) to improve it. The infection model was refined in Dutch volunteers [20], reaching stable egg counts that were comparable to what is seen in low-endemic regions. However, these Dutch volunteers originate from a non-endemic region and have never been exposed to hookworm.

It will be important to establish this model replicating the same procedure in Gabon to look at possible biological and immunological parameter changes that are exclusive to the SSA region, as has been observed during the Covid-19 pandemic [21]. These changes might account for varying efficacies of vaccines and drugs within the region in comparison to non-endemic regions. The development of a CHHI model in Africa will also allow for vaccine and drug development within the continent (see Table 2).

**Table 2:** The whys and hows of the controlled human infection model. Concepts particular to endemic regions are presented in bold

Why?	How
Need to develop and assess vaccine and drug candidates	• CHHI provides expedient assessment of vaccine and drug candidates • Allows for mining for vaccine candidates when combined with proteome and glycan arrays • **Creates a benchmark for assessing vaccine and drug efficacy in endemic populations**
Unwanted/allergic responses to candidate vaccines	• Report IgE reactivity to hookworm antigens • **De-risks future development of candidates by testing antibody recognition in hookworm endemic population (in particular testing for IgE recognition and eosinophilia with challenge material)**
Differing immune profiles between Africans and residents of non-endemic areas where vaccines are often developed	• Provides data on innate and adaptive cellular responses to hookworm infection • Report on IgM, IgG, IgG subclasses, and IgA reactivity to hookworm antigens • Provides insight into local (skin, airway mucosa, and gut) immune responses • **Allows for comparison between responses of volunteers from the Netherlands and Gabon**
Need to develop Correlates of protection	• Establish a profile of immune responses to hookworm as a surrogate of past exposure • **Using vaccine candidates, CHHI allows for the identification of molecular patterns that could correlate with protection**

(*CHHI*) Controlled human hookworm infection model

## Methods

As a first step towards establishing the Controlled Hookworm Human Infection model in a hookworm-endemic setting, we held a stakeholders meeting in Lambaréné, Gabon in November 2019, to identify key challenges and develop strategies to address them.

The stakeholders meeting addressed anticipated hurdles to the successful implementation of CHHI. Participants included representatives of Gabon’s Ministry of Health, the National Drug Authority, the National Ethics Committee, researchers and clinicians who manage hookworm infection and its complications, sociologists, community representatives, colleagues with experience of implementing controlled human schistosomiasis infections (CHI-S) from Uganda; and a team from Leiden University Medical Center, Leiden, The Netherlands who have experience in establishing the hookworm CHI.

## Results

### Regulatory and legislative considerations

There was a debate about the roles of different institutions and how they should interact in the legal process. It was agreed that the most appropriate way to bring this project to fruition will be to present a protocol or a detailed dossier to the ethics board. The ethics board will then review this and make recommendations both for the concerned institutions to improve the proposal and the Ministry of Health so that it may begin the groundwork for legislation and regulation with regards to CHHI in Gabon.

Another area that generated interest was whether existing legislation was robust enough to be adapted for innovative clinical research strategies that do not fall into the classical framework of vaccine trials. That is to say, whilst most clinical trials test a drug or vaccine that could protect or cure you, CHI intentionally infects volunteers before treating them. According to the Helsinki Accords, there must be legislation in place for any trials to take place. There was a suggestion that new legislation needs to be created to accommodate controlled human infection, but after extensive discussions, it was deemed not necessary to distinguish CHHI legislation from the legislation of clinical trials (see Table 3).

**Table 3:** Regulatory framework for ethics approval of CHHI in The Netherlands and Gabon, and CHI-S in Uganda

Country	Institutional and National Ethics review boards	Process of ethical approval for CHI studies	Legislation
The Netherlands	Universities have their own institutional ethics committees	Approval given by LUMC local medical ethics committee	Applicable law is the Wet medisch-wetenschappelijk Onderzoek in mensen (WMO): in English, Medical Research involving human subjects Act^a^
Gabon	Institutional review board (CERMEL) and National Ethics Committee (NEC)	Scientific approval from institution precedes submission for institutional ethics review. This is then followed by submission to the NEC	Under auspices of the Ministry of Health
Uganda	There are 23 Institutional Research Ethics Committee in Uganda. All these are accredited and regulated by the Uganda National Council for Science and Technology (UNCST)	Scientific approval from institution precedes submission for institutional ethics review. This is then followed by submission to the UNCST	No legislation or acts pertaining to human challenge studies exist in Uganda. Ministry of Health does not directly regulate research in Uganda

^a^ *CHHI is not formally a study with a medicinal product so European Medicines Agency regulations do not apply*

### Ethical considerations

At the ethical level, do no harm, the principle of non-maleficence, states one must avoid needless harm or injury that can arise through acts of commission or omission. The key consideration in this definition rests in the word ‘needless’; and whether or not it can be argued that in the case of CHHI, it is ‘needed’ to infect patients in order to achieve benefit. But there is understandable concern about infecting people in Africa for the purpose of medical advancement, even if there is a guaranteed cure.

This lies in a number of socio-political reasons. Historical atrocities involving deliberate infection of vulnerable populations have an important influence on thinking in this field [22]. And it should not be forgotten that western medicine and medical practitioners are viewed with a degree of scepticism in many parts of Africa. This was brought to light in the West African Ebola Outbreak when doctors were attacked and accused of spreading the virus [23]. Given this context, it is particularly important in Gabon to fully meet any ethical guidelines with regards to CHI. The challenge in African countries though is that these guidelines are either underdeveloped or do not exist. In this respect, the work being done on this project is pioneering within the Francophonie.

So, what might these ethical guidelines be? One area of discussion was whether to adopt international ethical frameworks, those that have been laid down in France or to pursue a Gabon-specific set of ethical criteria. Certainly, the principles articulated by the WHO in 2016 and benchmarks developed at the Malawi meeting on Controlled Human Infection Models in Low Income Countries could be employed to govern the ethical and regulatory approval process [24]. However, it was also noted that Gabon is one of a handful of African countries where the National Ethics Committee (NEC) operates as the sole arbitrator of ethical issues (unlike Uganda for example where many institutions have their own ethics committees that can influence policy) [25]. This, it was proposed, should be used to give credence to and bolster scientific and technological research. The discussions also touched on the subject of participant compensation, with the major question being how much to compensate. Compensation will cover transport, food, and any other costs incurred due to lost income.

### Socio-cultural considerations

The working group concluded that the social and cultural peculiarities of the communities in Gabon which will form the target group for CHI should be taken into consideration in the design of the study protocol. Culturally Gabon differs from most countries in Africa, in that there is less of a hierarchical structure within communities, and individual households are also key decision makers. Therefore, the traditional approaches such as contacting the head of the community as the key person to decide on the commencement of a study might not work in Gabon. In addition, some community beliefs such as being used as guinea pigs or the worry that researchers are there to kill them have to be countered through public enlightenment and awareness. Also there is a lack of exposure to western medicine in the rural areas as most people rely on their local traditions for healing. Changing such traditional beliefs and attitudes will require adequately educating the target population about the CHHI approach, and that practitioners also educate themselves about the target population. The same thought was re-iterated in a parallel workshop on the role of social sciences in clinical trials. The common conclusion drawn by both groups illustrates the need for close collaboration between the disciplines and the need for multi-sectoral approach to clinical trials to obtain the best possible results. Furthermore, a joint review meeting, with all regulatory authorities represented, was recommended, as well as engagement between the researchers and ethical and regulatory review bodies throughout the process of protocol development and implementation (see Table 4).

**Table 4:** Summary of topics discussed by workshop attendees with regards to the novelty of CHHI in Gabon and its appropriate implementation

Topics discussed during the workshop	Workshop proposals
Protocol development	• Regular consultations between researchers and interested regulatory authorities throughout process of protocol development and implementation • Protocol to be submitted to the Gabonese National Ethics Committee. NEC will then report to the Gabonese Ministry of Health • Study protocol and product dossier can be submitted as a single protocol document to the NEC • Develop a protocol that takes into consideration social and cultural peculiarities in Gabon
CHHI in a “vulnerable” population	• Develop pioneering national ethical guidelines for CHIM’s, in consultation with WHO recommendations, that will be strictly followed • Ensure full understanding of concept of CHI in communities where education level can be low
Appropriateness of current Gabonese legislation for the regulation of CHHI	• Existing legislation able to cover all aspects of CHHI • New legislation unnecessary
Media engagement	• Plan public engagement efforts that ensure the public can independently decide to or not to participate in CHHI in Gabon • Information management to be planned so the public is well informed and involved parties are prepared to alleviate undesired publicity

### Operational considerations

The operational considerations for the implementation of the Leiden CHHI model in Gabon include the establishment of donors from within the local population, the preparation of the L3 larvae locally in Gabon under GMP protocols at an “international standard”, and the creation of an adequately regulated hookworm laboratory dedicated to the manufacture of challenge material. This would involve research into locally acquired Gabonese *N. americanus* (*G-Na*) so that the exact strain of the challenge material is known, training of technical staff, development of infrastructure, trial runs in Gabon for verification of strict adherence to the procedural protocol, and the determination of the safest dose of challenge material to be used in the trial. A different option would be, to use the hookworm strain currently used at LUMC as challenge material, with samples being transferred from Leiden for final preparation of larvae in Gabon, eliminating the requirement for donation of parasites from infected Gabonese subjects.

In the LUMC model, an *N. americanus* originating from Madang, Papua New Guinea (PNG) is used as challenge material; however, the use of Gabonese *Na* (*G-Na*) is preferred in this case. Going forward, LUMC and Centre de Recherches Médicales de Lambaréné (CERMEL) staff will work in tandem to validate the LUMC model in Gabon, with the only difference being the use of the local strain. *G-Na* will need to be genotyped and characterized, and the genetic distance between PNG and *G-Na* strains identified alongside their relative safety and infectivity. Transfer of the Quality Assurance (QA) procedure already implemented at LUMC will be key. In addition, adoption of this QA would allow for capacity building in CERMEL, the local site.

A second option would be to transport hookworm-positive stool specimens from Leiden to Gabon, and then prepare the larvae in Gabon for challenge infection. As the time between larvae preparation and challenge infection is about 30 min, this procedure must be performed locally. The stool specimens can be shipped at ambient temperature, would have to arrive within 3–5 days from time of collection in Leiden. It will also require strictly following the International Air Transport Association (IATA) guidelines for the shipping of infectious materials. It will be important to work with customs officials and handling agents to ensure efficient release on arrival in Gabon. The risks of transport failure or errors during the preparation process would make the entire process uncertain and expensive. In addition, when patent infection of the PNG strain is achieved, a risk assessment will have to be made concerning the consequences of a possible introduction of the PNG strain into Gabonese soil. The consideration of an “in-patient” trial could be contemplated, and the costs of housing individuals for 12 weeks and compensation for containment for such a period of time would have to be budgeted for. Consultation and partnership with the Gabonese Agence Nationale des Parcs Nationaux is a must if such a scheme were to be adopted.

## Discussion

The establishment of CHHI in Gabon provides the opportunity to develop a regulatory, social, and ethical framework that suits Gabonese needs. The CHHI protocol will be expected to meet all standards of a Phase I trial. Years of experience with clinical trials at CERMEL [26], including challenge infections [13], mean that international standards for obtaining informed consent from participants will be adhered to. In anticipation of audits of the site, a quality control plan, data management plan and quality records will need to be available at all times.

In any vaccine trial that includes the study of disease prevention as an endpoint, participants would require treatment before vaccination. Depending on the number of participants involved and the ratio of infected vs uninfected in the community, this treatment may decrease community transmission to a point where the efficacy of a vaccine is difficult to measure or is measured inadequately. In this case it is especially applicable in regions where mass drug administration of anthelmintics is regularly practiced. CHHI in these regions will allow for identification of correlates of protection in individuals with previous exposure and background immune responses to hookworm infection, providing vital information for vaccine development and efficacy.

### Adaptation of the controlled human hookworm infection model in Gabon

The Leiden CHHI model has been rigorously tested to ensure high quality of the infectious agent, reproducibility, and the highest possible safety for the volunteers. The larval production is standardized in a GMP-like process before infective larvae are administered to volunteers. Larvae are cultured from chronically infected donors through a process involving multiple checks particularly to ensure *N. americanus* larvae are viable and clear of other pathogens. Motile larvae are dispensed onto gauzes and applied on the skin of the upper arms and legs of the volunteers. Twenty seven Dutch volunteers have been successfully infected using the model [20, 27]. No major safety concerns were identified, infection was reasonably well-tolerated by all participants. The well- developed protocols that work in LUMC with all safety precautions will be practiced in Gabon, and if needed changes will be made to adjust to the local situation. Healthy volunteers, with a good level of education, no intestinal helminth infections, and minimal risk of acquiring them during the trial period, will be selected. Following informed consent, it will be emphasized that they would need to adhere strictly to the procedure minimizing infection from outside of the trial. Volunteers will be followed up for 16 weeks and then treated with albendazole. Infection will be detected by microscopy and real-time PCR.

### Challenges

It will be difficult to find donors that are positive for *N. americanus* alone without coinfection with the helminths *Strongyloides stercoralis* or *A. duodenale* as coinfection rates are high. There is also a concern of hookworm transmission from a volunteer to others. All study participants will be counselled to practice good hygiene and to always defecate in a flush toilet/latrine. The study protocol must include measures to document toileting habits of participants.

It is important to consider that Gabon is a hookworm endemic region, therefore ensuring control over the infection given during the trial versus infection acquired naturally from the environment can be an important factor. Potentially, if the parasite is sufficiently genetically diverse, genetic markers could be used to confirm source of infection, but these can be costly.

## Conclusion

This will be the first attempt at establishing a CHHI model in Africa. These models have already been established in Europe where they have been improved in such a way that level of infection, measured as egg output, is not only stable but also reaches levels that represent what is found in endemic areas. Successful implementation and application of a CHHI model in Africa has the potential to address many of the roadblocks to the development of an effective vaccine for hookworm infection. Currently, controlled human infection models (CHIM) are seldom performed in Africa due to various social, ethical, infrastructural, and financial issues [24]. In this report we describe how these issues were thoroughly discussed at a forum between experts and local authorities, resulting in recommendations that fit international standards and attempt to match local expectations. The successful implementation of the CHHI model requires that the local context is taken into consideration, that public education targets the communities and authorities involved, and that community concerns are adequately addressed. The workshop is the first step in an iterative process that will call for deep commitment and continued collaboration between the stakeholders. We also aim at stimulating further interest in the implementation of this model in the sub-Saharan African setting.

## Supplementary Information

**Additional file 1.** Previous Controlled Human Hookworm Infection studies.

## Data Availability

Not applicable.ik

## Abbreviations

CERMEL: Centre de recherches médicales de lambaréné
CHHI: Controlled hookworm human infection
CHI: Controlled human infection
G-Na: Gabonese *Necator americanus*
LUMC: Leiden university medical center
NEC: National ethics committee
PNG: Papua New Guinea
QA: Quality assurance
SAC: School-aged children
