# Supplementary material for: Establishing a controlled hookworm human infection (CHHI) model for Africa: A report from the stakeholders meeting held in Lambaréné, Gabon, November 10–11, 2019
Source: Arch Public Health. 2021 Jul 5;79:120. doi: 10.1186/s13690-021-00650-z (PMC8256403; doi:10.1186/s13690-021-00650-z)
Supplement: Supplementary file 1 — Additional file 1. Previous Controlled Human Hookworm Infection studies. [file 13690_2021_650_MOESM1_ESM.docx]

Additional File 1

**Previous Controlled Human Hookworm Infection studies**

| **Study Population** | **N** | **Country** | **# Na-L3 applied** | **1^st^ time eggs observed in feces** | **Egg per gram feces (epg)** | **Adverse events** | **Reference** |
| --- | --- | --- | --- | --- | --- | --- | --- |
| **Healthy adults** | 29 | USA | 45 | 49-63 days | 72-268 | Skin rash; abdominal pain (50%), diarrhea. flatulence | [1] |
| **Healthy adults** | 5 | USA | 50 | 8-113 days | 500-1200 | Abdominal pain, flatulence | [2] |
| **Healthy adults** | 10 | UK | 10 (n=3)  25 (n-3)  50 (n-3), or  100 (n=1) | 4-6 weeks | Medians: 100 (10-25 larvae)- 700(50 larvae) | Skin rash, vomiting, diarrhea, abdominal pain | [3] |
| **Seasonal allergy patients** | 15 | UK | 10 | 4-6 weeks | >50 (4/15 negative) | Skin rash, indigestion, abdominal pan | [4] |
| **Celiac disease** | 17 | Australia | 10+5 | Not reported | Not reported (5/10 negative) | Skin rash, flatulence, abdominal pain | [5,6] |
| **Asthma** | 16 | UK | 10 | 6-8 weeks | 95-213 | Skin rash, gastrointestinal upset | [7] |
| **Crohn’s Disease** | 5 | Australia | 25-100 | 6-8 weeks | Not reported | Not reported | [8] |
| **Healthy Adult** | 1 | UK | 250 | 44-51 days | 800-4500 | Abdominal pain, diarrhea, nausea | [9] |
| **Seasonal allergy** | 1 | UK | 250 | Not reported | 3500-5000 | Not reported | [10] |
| **Healthy adult** | 1 | UK | 25-300 | Not reported | Not reported | Not reported | [11] |
| **Celiac Disease** | 12 | Australia | 20 (10 larvae administered twice 8 weeks apart) | Not reported | 120 EPG. (1 outlier with 724 EPG) | 2 subjects drawn after gluten micro challenge.  1 developed 3-cm blister (resolved without treatment)  1 had intermittent colic | [12] |
| **Celiac Disease** | 8 | Australia | 20 | Not reported | ? (all volunteers tested positive for hookworm) | Not reported | [13] |
| **Healthy adults** | 4 | Netherlands | 50 | 7-9 weeks | 40-2540 | Skin rash, itching, abdominal cramps | [14] |
| **Healthy adults** | 20 | USA | 25 (n=10)  50 (n=10) | 7 weeks | Medians: 0 (25 larvae), 0-33 (50 larvae) | Skin rash, flatulence, abdominal bloating, abdominal pain | [15] |
| **Healthy adults** | 23 | Netherlands | A: 50 (n=8)  B: 100 (n=7)  C: 150 (n=8) | A: 5-8 weeks  B: 7-9 weeks  C: 7-9 weeks | Medians (12-16 weeks after (first) infection):  A: 1010  B:1440  C: 1270 | Skin rash, itching, abdominal cramps, flatulence, nausea, diarrhea | [16] |

**References**

[1] Cline BL, Little MD, Bartholomew RK, Halsey NA. Larvicidal activity of albendazole against Necator americanus in human volunteers. Am J Trop Med Hyg 1984;33:387–94. https://doi.org/10.4269/ajtmh.1984.33.387.

[2] The clinical and immunologic responses of normal human volunteers to low dose hookworm (Necator americanus) infection. - PubMed - NCBI n.d. https://www.ncbi.nlm.nih.gov/pubmed/3605493 (accessed February 17, 2020).

[3] Mortimer K, Brown A, Feary J, Jagger C, Lewis S, Antoniak M, et al. Dose-ranging study for trials of therapeutic infection with Necator americanus in humans. Am J Trop Med Hyg 2006;75:914–20.

[4] Feary J, Venn A, Brown A, Hooi D, Falcone FH, Mortimer K, et al. Safety of hookworm infection in individuals with measurable airway responsiveness: a randomized placebo-controlled feasibility study. Clin Exp Allergy 2009;39:1060–8. https://doi.org/10.1111/j.1365-2222.2009.03187.x.

[5] Daveson AJ, Jones DM, Gaze S, McSorley H, Clouston A, Pascoe A, et al. Effect of hookworm infection on wheat challenge in celiac disease--a randomised double-blinded placebo controlled trial. PLoS ONE 2011;6:e17366. https://doi.org/10.1371/journal.pone.0017366.

[6] pubmeddev, al MH et. Suppression of inflammatory immune responses in celiac disease by experimental hookworm infection. - PubMed - NCBI n.d. https://www.ncbi.nlm.nih.gov/pubmed/21949691 (accessed February 17, 2020).

[7] Feary JR, Venn AJ, Mortimer K, Brown AP, Hooi D, Falcone FH, et al. Experimental hookworm infection: a randomized placebo-controlled trial in asthma. Clin Exp Allergy 2010;40:299–306. https://doi.org/10.1111/j.1365-2222.2009.03433.x.

[8] A proof of concept study establishing Necator americanus in Crohn’s patients and reservoir donors. - PubMed - NCBI n.d. https://www.ncbi.nlm.nih.gov/pubmed/16344586 (accessed February 27, 2020).

[9] Ogilvie BM, Bartlett A, Godfrey RC, Turton JA, Worms MJ, Yeates RA. Antibody responses in self-infections with Necator americanus. Trans R Soc Trop Med Hyg 1978;72:66–71. https://doi.org/10.1016/0035-9203(78)90303-6.

[10] Turton JA. Letter: IgE, parasites, and allergy. Lancet 1976;2:686. https://doi.org/10.1016/s0140-6736(76)92492-2.

[11] Ball P a. J, Bartlett A. Serological reactions to infection with Necator americanus. Trans R Soc Trop Med Hyg 1969;63:362–9. https://doi.org/10.1016/0035-9203(69)90011-X.

[12] pubmeddev, al CJ et. Experimental hookworm infection and gluten microchallenge promote tolerance in celiac disease. - PubMed - NCBI n.d. https://www.ncbi.nlm.nih.gov/pubmed/25248819 (accessed February 17, 2020).

[13] Cantacessi C, Giacomin P, Croese J, Zakrzewski M, Sotillo J, McCann L, et al. Impact of experimental hookworm infection on the human gut microbiota. J Infect Dis 2014;210:1431–4. https://doi.org/10.1093/infdis/jiu256.

[14] Hoogerwerf M-A, Coffeng LE, Brienen EAT, Janse JJ, Langenberg MCC, Kruize YCM, et al. New Insights Into the Kinetics and Variability of Egg Excretion in Controlled Human Hookworm Infections. J Infect Dis 2019;220:1044–8. https://doi.org/10.1093/infdis/jiz218.

[15] Diemert D, Campbell D, Brelsford J, Leasure C, Li G, Peng J, et al. Controlled Human Hookworm Infection: Accelerating Human Hookworm Vaccine Development. Open Forum Infect Dis 2018;5:ofy083. https://doi.org/10.1093/ofid/ofy083.

[16] Hoogerwerf M-A, Koopman JPR, Janse JJ, Langenberg MCC, van Schuijlenburg R, Kruize YCM, et al. A randomized controlled trial to investigate safety and variability of egg excretion after repeated controlled human hookworm infection. J Infect Dis n.d. https://doi.org/10.1093/infdis/jiaa414.
